# Determination of Cytotoxic Activity of Selected Isoquinoline Alkaloids and Plant Extracts Obtained from Various Parts of *Mahonia aquifolium* Collected in Various Vegetation Seasons

**DOI:** 10.3390/molecules26040816

**Published:** 2021-02-04

**Authors:** Tomasz Tuzimski, Anna Petruczynik, Barbara Kaproń, Anna Makuch-Kocka, Małgorzata Szultka-Młyńska, Justyna Misiurek, Grażyna Szymczak, Bogusław Buszewski

**Affiliations:** 1 Department of Physical Chemistry, Faculty of Pharmacy, Medical University of Lublin, Chodźki 4a, 20-093 Lublin, Poland; 2 Department of Inorganic Chemistry, Faculty of Pharmacy, Medical University of Lublin, Chodźki 4a, 20-093 Lublin, Poland; justyna.misiurek@umlub.pl; 3 Department of Clinical Genetics, Faculty of Medicine, Medical University of Lublin, Radziwiłłowska 11, 20-080 Lublin, Poland; barbara.kapron@umlub.pl; 4 Department of Pharmacology, Faculty of Health Sciences, Medical University of Lublin, Chodźki 4a, 20-093 Lublin, Poland; anna.makuch-kocka@umlub.pl; 5 Department of Environmental Chemistry and Bioanalytics, Faculty of Chemistry, Nicolaus Copernicus University, Gagarina 7, PL-87-100 Torun, Poland; szultka.malgorzata@wp.pl (M.S.-M.); bbusz@chem.umk.pl (B.B.); 6 Botanical Garden of Maria Curie-Skłodowska, University in Lublin, Sławinkowska 3, 20-810 Lublin, Poland; grazyna.szymczak@poczta.umcs.lublin.pl

**Keywords:** cytotoxic activity, HPLC-DAD, LC-MS/MS, isoquinoline alkaloids, *Mahonia aquifolium*

## Abstract

Melanoma is a serious form of skin cancer that begins in cells known as melanocytes. While it is less common than the other forms of skin cancer, melanoma is more dangerous because of its ability to spread to other organs more rapidly if it is not treated at an early stage. The number of people diagnosed with melanoma has increased over the last few decades. The most widely used treatments include surgery, chemotherapy, and radiation therapy. The search for new drugs to treat various cancers is one of the most important challenges of modern scientific research. Some isoquinoline alkaloids found in different plant species have strong cytotoxic effects on various cancer cells. We tested the effect of isoquinoline alkaloids and extracts obtained from various parts of *Mahonia aquifolium* collected in various vegetation seasons on human melanoma cancer cells and our data indicated that investigated extract induced significant reduction in cell viability of Human malignant melanoma cells (A375), human Caucasian malignant melanoma cell line (G361), and human malignant melanoma cell line (SKMEL3 cancer cell lines in a dose- and time-dependent manner. Differences in cytotoxic activity were observed for extracts obtained from various parts of *Mahonia aquifolium*. Significant differences were also obtained in the alkaloids content and cytotoxic activity of the extracts depending on the season of collection of plant material. Our investigations exhibit that these plant extracts can be recommended for further in vivo experiments in order to confirm the possibility of their use in the treatment of human melanomas.

## 1. Introduction

*Mahonia* is an evergreen shrub plant of the family Berberidaceae and includes species that are native to Eastern Asia, North America, and Central America. Plants of genus *Mahonia* exhibit antibacterial, antifungal, and anti-inflammatory properties and are widely used in folk medicine worldwide as a cure for tuberculosis, dysentery, pharyngolaryngitis, eczema, and different skin disorders [1]. Different organs of *Mahonia* has various biological activities [2]. Some *Mahonia* species have cytotoxic activity against various human cancer cells [1]. Currently, searching for antitumor agents is the one of the most important in the development of new drugs. Various plants produce many secondary metabolites to treat cancer. Herbal medicines exhibit anticancer effects by exciting DNA repair mechanism, inducing antioxidant action, retarding cancer inducing hormones and enzymes boosting the construction of defensive enzymes, and enhancing immunity [3]. Godevac et al. investigated cytotoxic metabolites from *Mahonia aquifolium* using ^1^H NMR-based metabolomics method [1]. Isoquinoline alkaloids constitute a major class of compounds isolated from the genus *Mahonia* and they are responsible for many properties of plants of this genus. The following isoquinoline alkaloids, namely, berberine, jatrorrhizine, palmatine, magnoflorine, isocorydine, and oxyacanthine, were previously isolated from various *Mahonia* species. Berberine, palmatine, and berbamine were identified as the components with the highest cytotoxic activity.

Berberine is main cytotoxic active ingredients of *Mahonia aquifolium* and the compound is known to have an inhibitory effect on various cancer cell lines. Berberine and its various synthetic analogues exhibit anticancer activity against, e.g., cervical, ovarian, and endometrial cancers [4], cervical cancer [5], gastric cancer [6], breast cancer [7], colorectal cancer [8], esophageal carcinoma [9], lung adenocarcinoma [10], and leukemia [11]. Berberine was also tested for inhibition of melanoma cancer cells, e.g., mouse melanoma B16 cells [12], B16F10 cells [13], human melanoma A375 cell line [14], human melanoma A375.S2 and A375.S2/PLX cells [15]. Palmatine and its analogues also possessed anticancer activity against, e.g., 7701QGY, SMMC7721, HepG2, CEM, CEM/VCR, KIII, Lewis cell lines [16], MCF-7 (breast cancer) [17]. Magnoflorine anticancer activity was investigated against, e.g., NCI-H1299 lung, MDA-MB-468 breast, T98G glioma, and TE671 rhabdomyosarcoma cancer cells [18].

For determination of isoquinoline alkaloids contents in various *Mahonia* species extracts high performance liquid chromatography (HPLC) was often applied [19]. Most often analysis were performed on octadecyl (C18) columns with mobile phases contained organic modifier (most often acetonitrile or methanol), water and addition of acids, e.g., phosphoric acid [19], formic acid [20,21], buffer at acidic pH [22], and addition of salts, e.g., sodium dihydrogen phosphate [2]. Capillary high performance liquid chromatography was also applied for analysis of alkaloids from *Mahonia aquifolium* extract [23]. For this purpose capillary C18 column and mobile phase containing methanol, water and formic acid was used. Various detection mode were applied for detection of alkaloids in *Mahonia* species. Most often diode array detection (DAD) [19,22], mass spectrometry (MS) or tandem mass spectrometry (MS/MS) [20], DAD, and MS/MS [23] were used for this purpose. The application of various detection methods allowed the detection of alkaloids in extracts at different levels of their concentrations. Wang et al. determined columbamine, jatrorrhizine, palmatine, and berberine in *Mahoniae Caulis* by HPLC-DAD [22]. Separation was performed on C18 column with mobile phase containing acetonitrile and phosphate buffer at pH 3.0. LOQ values obtained by the procedure were from 2.2 µg/mL for berberine to 3.6 µg/mL for jatrorrhizine. For separation of alkaloids in extracts obtained from *Mahonia leschenaultia* and *Mahonia napaulensis* roots by ultra-high-performance liquid chromatography-electrospray ionization-tandem mass spectrometry (UHPLC–ESI–MS/MS), C18 column and mobile phase acetonitrile, water, and formic acid were applied [20]. Obtained for investigated alkaloids LOQ values were from 0.24 to 1.46 ng/mL. However, the main alkaloids responsible for the biological activity of *Mahonia* extracts they are found in high contents and can be successfully determined using various detection techniques.

The cytotoxic properties of *Mahonia* species extracts have been rarely investigated. The investigations was conducted only against a few cell lines. Cytotoxicity of extracts obtained from *Mahonia aquifolium* was in vitro tested against human cervical adenocarcinoma cell line (HeLa) [1]. All examined extracts showed moderate activity against the cell line. Authors correlated chemical composition of the *Mahonia aquifolium* extracts determined by a^1^H NMR-based metabolomics method, with the results of cytotoxic activity testing. Protoberberine alkaloids berberine and palmatine and bisbenzyliso-quinoline alkaloid berbamine were identified as compounds with the highest cytotoxic activity against HeLa cell line. Cytotoxicity, apoptosis, and cell cycle arrest induction by extracts obtained from *Mahonia fortune* was investigated on cancer cell lines: breast cancer cells (MCF-7), epidermal cell line (A431), and glioma cell line (U87-MG) [24]. Investigated extracts exhibited cytotoxicity effect on cancer cells higher than normal cells. Latha et al. observed a strong anticancer activity of methanol root extract obtained from *Mahonia leschenaultii* in suppressing Dalton’s ascitic lymphoma cancer cell growth in a mouse model by controlling haematological, lipid, serum enzymes, and other derived parameters effectively [3]. Water extract of leaves of *Mahonia bealei* inhibited the proliferation of human colon cancer (HT-29) cells [25]. The in vitro and in vivo apoptotic effects of *Mahonia oiwakensis* on human lung cancer cells was investigated by Wong et al. [26].

The aim of this work was investigations of alkaloid compositions of plant extracts obtained from various parts of *Mahonia aquifolium* collected in various vegetation seasons by HPLC-DAD as well as HPLC-MS/MS. Anticancer activities of different isoquinoline alkaloids and plant extracts containing these alkaloids against Human malignant melanoma cells (A375), human Caucasian malignant melanoma cell line (G361), and human malignant melanoma cell line (SKMEL3 cancer cell lines were also investigated) These extracts have not been previously tested against these cancer cell lines.

## 2. Results and Discussion

### 2.1. HPLC-DAD and LC-MS/MS Analysis of Plant Extracts

**Figure 1:**
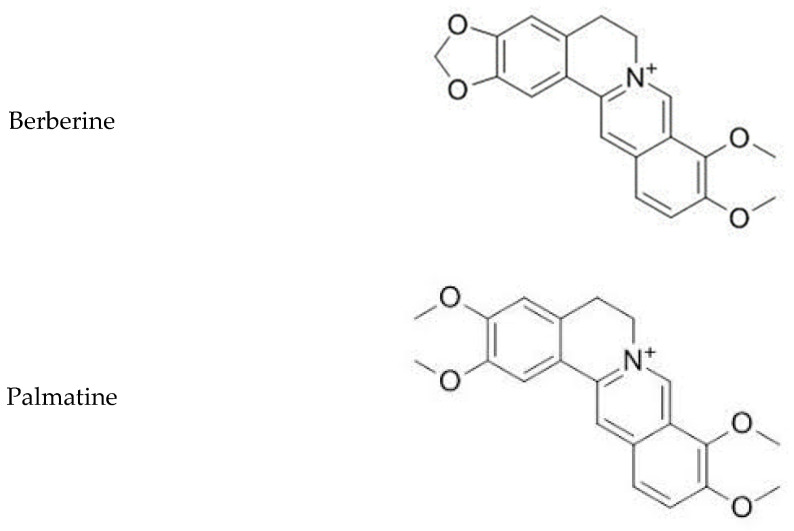

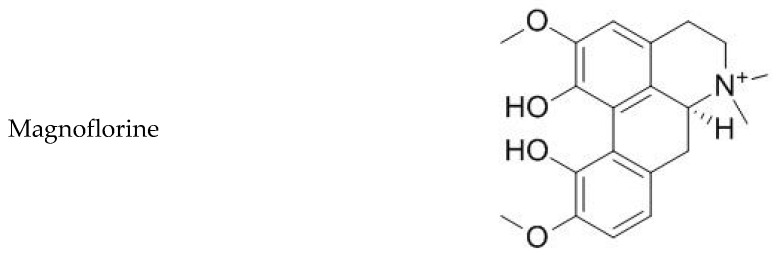
Chemical structures of investigated isoquinoline alkaloids.

Alkaloid standards (see Figure 1) were chromatographed on Polar RP 18 column in eluent system containing acetonitrile, water and 1-butyl-3-methylimidazolium tetrafluoroborate. The chromatographic condition was based on the previously published method applied for determination of isoquinoline alkaloids [27] after appropriate modification. Retention times for investigated alkaloid standards are presented in Table 1. The quantitative analysis was performed by a calibration curve method. Calibration curves were constructed by analyzing the samples at eight concentrations, ranging from 0.001 to 0.2 mg/mL (Table 1).

**Figure 2:**
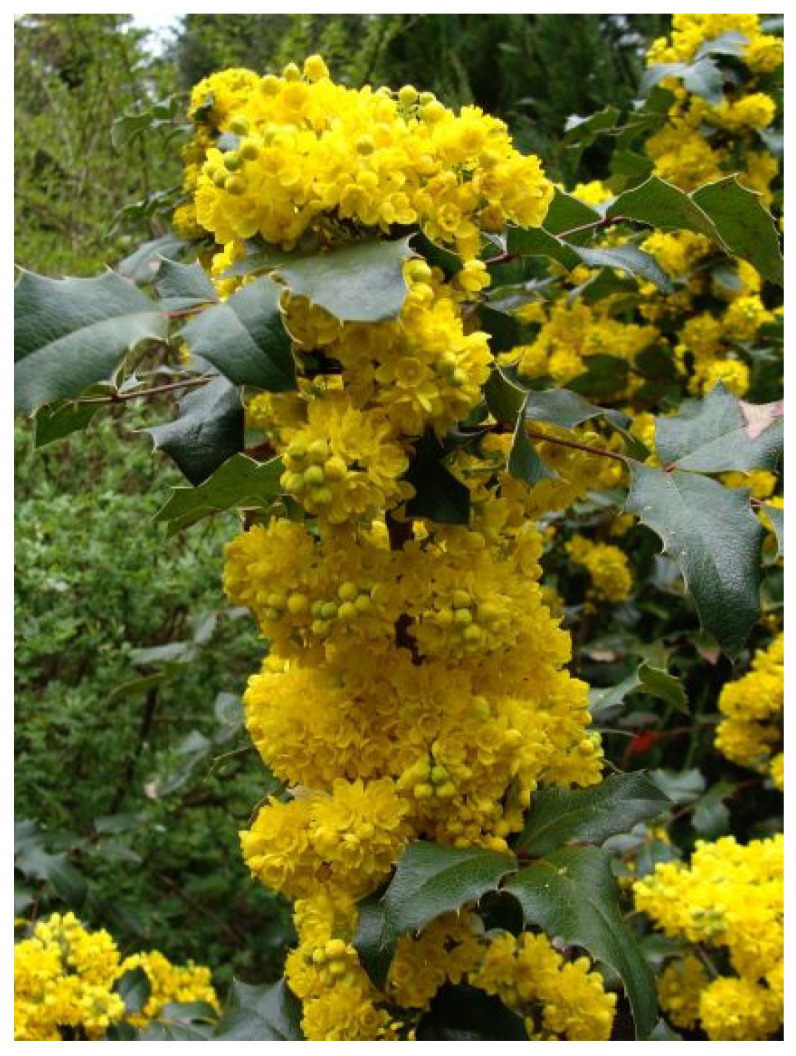
Photograph of *Mahonia aquifolium* under flowering. (The photo comes from the archives of the Botanical Garden of Maria Curie-Skłodowska University in Lublin (Poland)).

In the next step of experiments alkaloid contents were determined in extracts obtained from *Mahonia aquifolium* (Figure 2).

**Figure 3:**
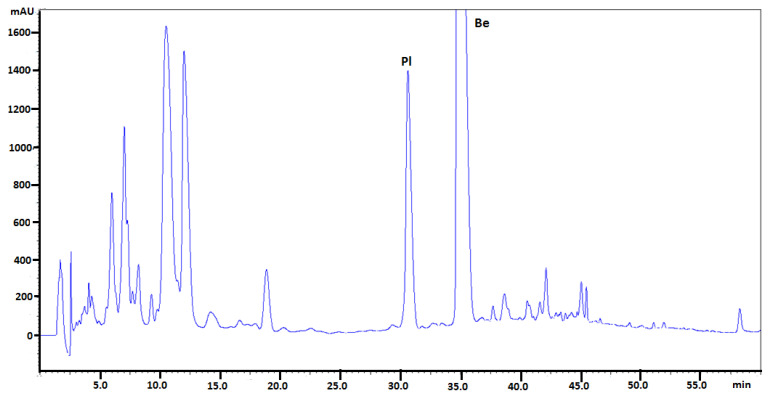
Chromatogram obtained for *Mahonia aquifolium* cortex extract obtained on Polar Reversed Phase (Polar RP) column with mobile phase MeCN, water and 1-butyl-3-methylimidazolium tetrafluoroborate. Abbreviations: Pl—palmatine, Be—berberine. Plant material was collected during flowering.

**Figure 4:**
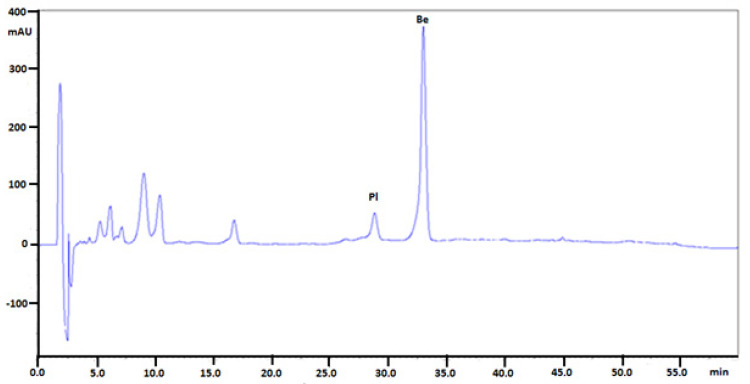
Chromatogram obtained for *Mahonia aquifolium* roots extract (the extract was diluted 50 times) obtained on Polar RP column with mobile phase MeCN, water and 1-butyl-3-methylimidazolium tetrafluoroborate. Abbreviations: Pl—palmatine, Be—berberine. Plant material was collected during flowering.

**Figure 5:**
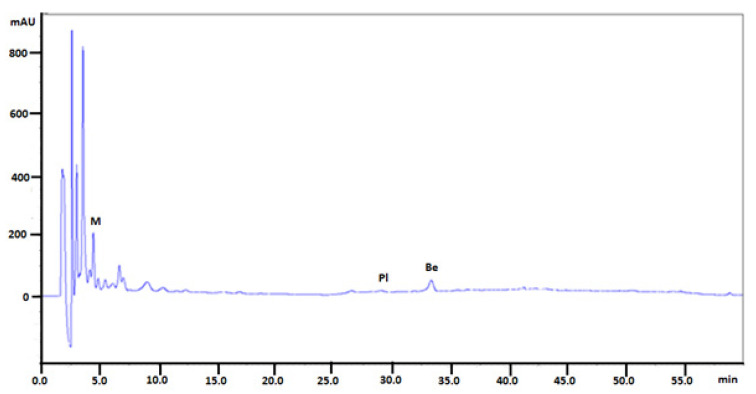
Chromatogram obtained for *Mahonia aquifolium* leaves extract obtained on Polar RP column with mobile phase MeCN, water and 1-butyl-3-methylimidazolium tetrafluoroborate. Abbreviations: Pl—palmatine, Be—berberine, M—magnoflorine. Plant material was collected during flowering.

The quantitative determination of selected isoquinoline alkaloids in plant extracts were performed in the same chromatographic conditions. Examples of obtained chromatograms are presented in Figure 3, Figure 4 and Figure 5.

**Figure 6:**
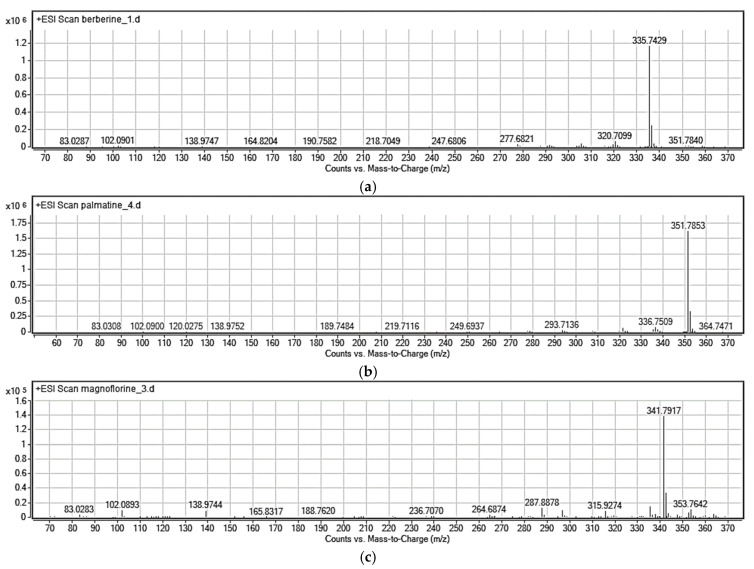
Representative MS spectra (**a**–**c**) for berberine (cortex), palmatine (root) and magnoflorine (cortex) obtained for studied isoquinoline alkaloids from *Mahonia aquifolium* extracts. Plant material collected during flowering. Experimental conditions: mobile phase composition: 70% *v*/*v* 0.1% HCOOH, 30% *v*/*v* MeCN, flow rate: 0.6 mL/min, autosampler temperature 20 °C, drying gas flow rate 7.0 L/min; shielding gas flow rate 5.0 L/min; nebulizer gas pressure 40 psi; skimmer voltage 50 V, octopole voltage 800 V; capillary voltage 3500 V; drying gas temperature 295 °C; shielding gas temperature 315 °C; fragmentor voltage 195 V.

**Figure 7:**
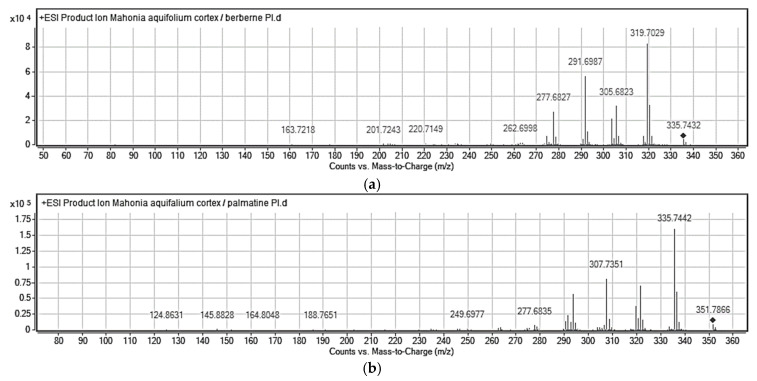

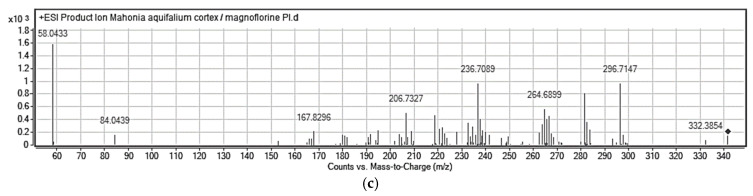
Representative product ion spectra MS/MS (**a**–**c**) for berberine (cortex), palmatine (root) and magnoflorine (cortex) obtained for studied isoquinoline alkaloids from *Mahonia aquifolium* extracts. Plant material collected during flowering. Mobile phase composition: *v*/*v* 0.1% HCOOH, 30% MeCN *v*/*v*. Experimental conditions: flow rate 0.6 mL/min, autosampler temperature 20 °C, drying gas flow rate 7.0 L/min; shielding gas flow rate 5.0 L/min; nebulizer gas pressure 40 psi; skimmer voltage 50 V, octopole voltage 800 V; capillary voltage 3500 V; drying gas temperature 295 °C; shielding gas temperature 315 °C; fragmentor voltage 195 V; CID energy in both MS and MS/MS were 5 and 20 eV.

The identities of the analyte peaks in plant extracts were confirmed by the comparison of their retention times and UV spectra with the retention times and MS and product ion MS/MS spectra of relevant alkaloid standards. Isoquinoline alkaloids were quantified in extracts obtained from various parts of *Mahonia aquifolium* collected in various vegetation seasons. Relevant isoquinoline alkaloids were identified based on MS spectra for berberine (*m*/*z* = 335.7429), magnoflorine (*m*/*z* = 341.7917) and palmatine (*m*/*z* = 351.7853), respectively. Moreover, the presence of them in real plant samples regarding different morphological parts (barked stalk, cortex, fruit, leaves, roots before, after or during flowering) was confirmed by MS/MS spectra. Representative MS and MS/MS spectra obtained for alkaloids from extracts are presented in Figure 6 and Figure 7.

The identification of studied alkaloids in the plant extracts was based on the accurate mass measurements, the isotopic distribution of parent ions, and the study of their fragmentation patterns.

In the MS/MS spectrum of berberine (Figure 6a), the major product ion appear at *m*/*z* = 319.7029 and correspond to elimination of methyl radical and CH_4_ respectively from the methoxy substituent. The ion at *m*/*z* = 305.6823 is formed by continuous elimination of two methyl radicals and the ion at *m*/*z* = 304.1893 is formed by loss of CH_3_OH from the precursor ion. The ions at *m*/*z* = 291.6987 and 277.6827 are then formed by loss of CO from the ions at *m*/*z* = 319.7029 and *m*/*z* = 305.6823, respectively. Moreover, this sequential loss of a methyl radical and CO is the characteristic fragmentation pathway of this kind of alkaloid [28]. This fragmentation behavior also occurs in other alkaloids such as palmatine. Additionally, the MS/MS spectra of [M + H]^+^ ions for palmatine are characterized by the presence of the most abundant ion at *m*/*z* = 335.7442 and several fragment ions, as shown in Figure 6b. These characteristic ions at *m*/*z* = 307.7351, 277.6835 and 244.6977 were formed mainly by the cleavage of substituted methoxyl and methylenedioxyl groups on the A- and D-rings, respectively. In case of magnoflorine the signal at *m*/*z* = 296.7147 confirms the loss of two methyl groups and NH group at the quaternary ammonium ion. Moreover, the signal at *m*/*z* = 264.6899 confirms the detachment of additional –CH_3_OH group out of the signal at *m*/*z* = 296.7147.

Great differences in the contents of investigated isoquinoline alkaloids have been observed not only in various plant parts and also in the same part of plant collected in various vegetation seasons (Table 2). The highest concentration of berberine was determined in roots and cortex collected during flowering (7.036 and 3.313 mg/g of dry plant material, respectively). The contents of the alkaloid were significantly lower before and after flowering (only 0.133 and 0.066 mg/g of dry plant material, respectively). The high content of berberine was also identified in barked stalk during flowering (0.436 mg/g of dry plant material). In other part of *Mahonia aquifolium* lower contents of the alkaloid were observed. The highest content of columbamine was also determined in cortex collected during flowering (0.730 mg/g of dry plant material). Higher contents of magnoflorine were detected in leaves before and after flowering and were about 0.32 mg/g of dry plant material, while in leaves collected during flowering 0.2342 mg of magnoflorine in g of dry plant material was determined. Except in the leaves, magnoflorine in smaller amounts was identified in cortex collected before and after flowering and in barked stalk collected after flowering. The highest content of palmatine was observed in cortex collected during or after flowering (about 0.79 mg/g of dry plant material). High palmatine content was also found in barked stalk and also roots collected after flowering (0.79 and 0.62 mg/g of dry plant material, respectively). Only very small contents of berberine and columbamine (<LOQ) were found in *Mahonia aquifolium* fruit extract. The highest content of berberine was found in roots during flowering, palmatine in the cortex collected during the flowering of the plant, while the highest content of magnoflorine was identified in the leaves collected after flowering. Both in the roots and the cortex a significant increase in berberine and palmatine contents during the flowering of *M. aquifolium* was observed. The investigations show that the best season to collecting the plant material (roots and cortex) in order to obtain the highest content of isoquinoline alkaloids with high cytotoxic activity (berberine and palmatine) is during flowering. While, in order to obtain the plant material with the higher content of magnoflorine, the leaves should be collected before or after flowering.

### 2.2. Investigation of In Vitro Anticancer Activity of Isoquinoline Alkaloid Standards

**Figure 8:**
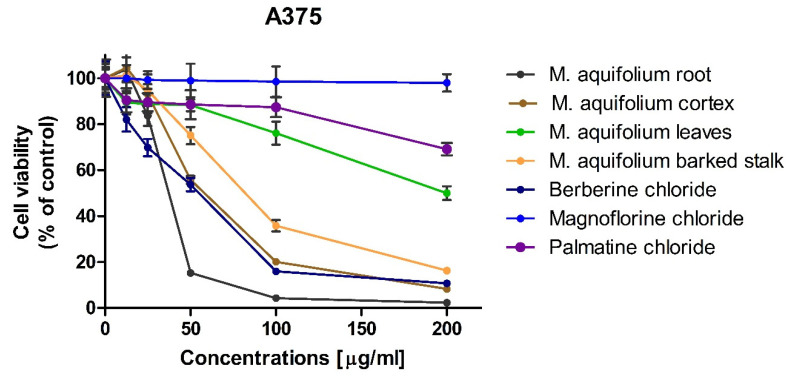
Effect of alkaloid standards and *Mahonia aquifolium* extracts on the viability of human melanoma A375 cells.

**Figure 9:**
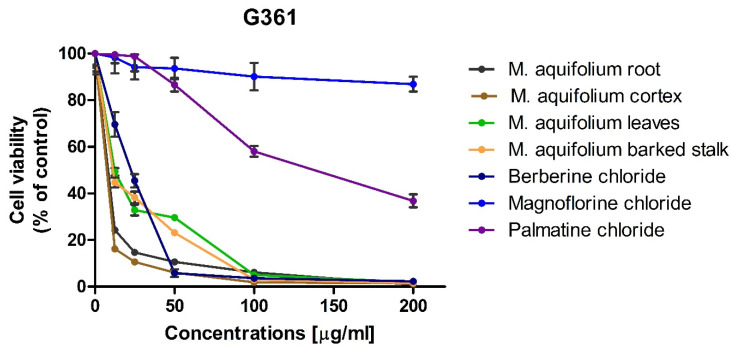
Effect of alkaloid standards and *Mahonia aquifolium* extracts on the viability of human melanoma G361 cells.

**Figure 10:**
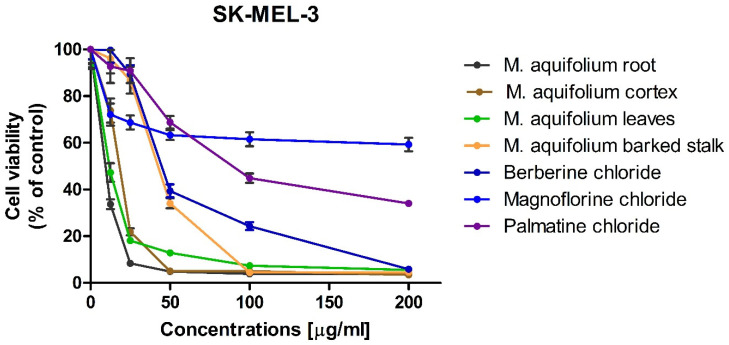
Effect of alkaloid standards and *Mahonia aquifolium* extracts on the viability of human melanoma SK-MEL-3 cells.

The cytotoxic activity of alkaloid standards: berberine, magnoflorine and palmatine were carried out using three melanoma cell lines (i.e., A375, G-361, and SK-MEL-3) that differ in origin and mutated genes. The effect of alkaloid standards and *Mahonia aquifolium* extracts on the viability of A375, G-361, and SK-MEL-3 cells are presented in Figure 8, Figure 9 and Figure 10. Results were reported as the percentage of relative viability of the treated cells when compared to the untreated control cells. Magnoflorine exhibited the lowest cytotoxic activity against all tested cancer cells. At the highest concentration tested (200 µg/mL), magnoflorine decreased the viability of the melanoma cells by 2% (A375), 13.12% (G-361), and 40.74% (SK-MEL-3). Much higher cytotoxic activity was observed for palmatine. Among the investigated melanoma cell lines, A375 cells turned out to be the least sensitive to this alkaloid (viability > 70% at the concentration of 200 µg/mL). Viability of the other two cell lines was about 40% (vs. untreated cells) after treating by palmatine (200 µg/mL). The highest cytotoxic effect towards all tested cell lines was observed for berberine. It inhibited G361 cells growth by more than 90% as soon as its concentration in culture medium reaches at least 50 µg/mL. Sensitivity of the other two cell lines (A375 and SK-MEL-3) against berberine was slightly slower; however, it still reduced A375 cells viability by more than 80% (when administered at the concentration not less than 100 µg/mL) and inhibited the growth of SK-MEL-3 cells by more than 90% at the highest concentration tested.

IC_50_ values, of the investigated alkaloid standards against three melanoma cell lines were also calculated (Table 3). IC_50_ for magnoflorine were higher than 200 µg/mL for all tested cell lines. IC_50_ obtained for palmatine against A375cells but against G361 and SK-MEL-3 cell lines these values were about 120 and 88 µg/mL what indicated on moderate cytotoxic activity. The lowest values of IC_50_ were obtained for berberine (from 21.25 against G361 to 52.73 µg/mL against A375 cell lines). Berberine exhibited the higher cytotoxic activity against all tested melanoma cell lines. It showed moderate cytotoxic activity in range between 20 and 200 μg/mL against all tested melanoma cell line. IC_50_ obtained for palmatine indicated on moderate cytotoxicity against G361 and SK-MEL-3 cells and weak cytotoxicity against A375 cells (IC_50_ > 200 µg/mL. Magnoflorine exhibit low cytotoxic activity against all tested melanoma cell lines.

### 2.3. Investigation of In Vitro Anticancer Activity of Plant Extracts

In vitro cytotoxic activity of the investigated plant extracts was examined against the same melanoma cell lines as the previously investigated alkaloid standards. Since the highest concentrations of alkaloids were determined in extracts obtained during flowering, these extracts were selected for cytotoxic activity evaluation. High cytotoxicity of all of the investigated *Mahonia aquifolium* extracts against A375, G-361, and SK-MEL-3 melanoma cells was observed. However, out of the investigated melanoma cell lines, A375 cells exhibited the lowest sensitivity to *Mahonia aquifolium* extract, similarly as to the previously tested alkaloid standards. Nevertheless, the extract from *Mahonia aquifolium* root almost completely inhibited the growth of A375 at the concentrations of 100 and 200 µg/mL (melanoma cells viability equaled 4.27 and 2.28%, respectively). Significantly higher cytotoxicity, even at the lower concentrations of the extracts, was observed against human melanoma G-361 and SK-MEL-3 cell lines. Viability of G-361 cells was as low as 16.18 and 24.27% when exposed to the lowest concentration (12.5 µg/mL) of extracts obtained from cortex and root of *M. aquifolium*, respectively. At the same concentration, extracts obtained from leaves and barked stalk decreased the viability of G-361 cells to about 50%. Further increasing of extract concentrations resulted in a dose-dependent inhibition of melanoma cells viability. At the highest concentration tested (i.e., 200 µg/mL), viability of G-361 cells was below 2%. Different response to the investigated extracts was observed for SK-MEL-3 cells. Similarly as in the case of melanoma G-361 cells, the highest cytotoxic activity was observed for extract from *M. aquifolium* root. However, the extract from *M. aquifolium* leaves in a concentration of 12.5 µg/mL reduced the viability of SK-MEL-3 cells only slightly less effectively than extract from roots. The effect of *M. aquifolium* cortex and barked stalk extracts, at the concentration of 12.5 µg/mL, was weaker while the viability of SK-MEL-3 cells decreased to 73.86 and 96.16%, respectively. However, at the concentrations higher or equal to 50 µg/mL, extracts from roots and cortex of *M. aquifolium* were more active again. At the mentioned concentration (i.e., 50 µg/mL), they reduced the growth of SK-MEL-3 cells to 4.91 and 5.06%.

IC_50_ values were also determined for *M. aquifolium* extracts (Table 3). In almost all cases IC_50_ values were lowest than these obtained for investigated alkaloid standards including berberine with the highest cytotoxicity. Only extracts obtained from *M. aquifolium* leaves and barked stalk IC_50_ values were highest than IC_50_ obtained for berberine. The highest activity against A375 cells was observed after treating by the extract obtained from root (IC_50_ = 34.13 µg of dry plant extract/mL). The highest activity against G361 exhibited extract obtained from *M. aquifolium* cortex (IC_50_ = 4.78 µg of dry plant extract/mL). The most cytotoxic against SK-MEL-3 cell line was extract obtained from root (IC_50_ = 10.53 µg of dry plant extract/mL). In all cases IC_50_ values obtained for plant extracts were lower than those obtained for alkaloid standards. This may indicate on the synergism of the action of these alkaloids as well as the influence on the cytotoxic activity of other extract components, not determined in our research. The results obtained for *M. aquifolium* extracts clearly indicate on a very high potential of cytotoxic properties of these extracts against tested human melanoma cells. Foe almost all extracts obtained IC_50_ values indicated on high or moderate cytotoxic activity. Only for *M. aquifolium* leaves extract against A375 cells IC_50_ value was higher than 200 µg/mL. Extracts obtained from roots and cortex showed high cytotoxic activity against G361 and SK-MEL-3 cell lines and moderate activity against A375 cells. It indicated that *M. aquifolium* extracts, especially from roots and cortex have good anticancer potential and suggested that these extracts should be subjects of further investigations as potential cancer therapeutics.

Extracts of *M. aquifolium* especially obtained from roots and cortex exhibited significantly lower IC_50_ values compared to IC_50_ obtained for various plant extracts described as inhibitors of melanoma cells viability. For example IC_50_ values obtained for murine B16 melanoma 4A5 cells treated by *Anastatica hierochuntica* methanolic and ethanolic extracts were 100 µg/mL and 60 µg/mL, respectively [30]. IC_50_ for B16F10 melanoma cells treated by *Pueraria thunbergiana* extracts obtained from different parts of plant were from 213.93 to 2010.34 µg/mL [31]. For A375 human melanoma cell line after treating by extract from *Daucus virgatus* IC_50_ about 300 µg/mL was obtained [32]. Extracts from *Tabernaemontana catharinensis* containing indole alkaloids showed toxicity against A37 cells with IC_50_ from 11.73 to 138.54 µg/mL [33]. IC_50_ = 19.98 µg/mL was obtained on B16F10 with *Solanum lycocarpum* extract [34]. IC_50_ obtained for B16F10 cells with leaf extract from *Indigofera hirsuta* L. was 80.9 µg/mL [35].

### 2.4. Correlation of Alkaloid Contents with Cytotoxic Activity of M. aquifolium Extracts

**Figure 11:**
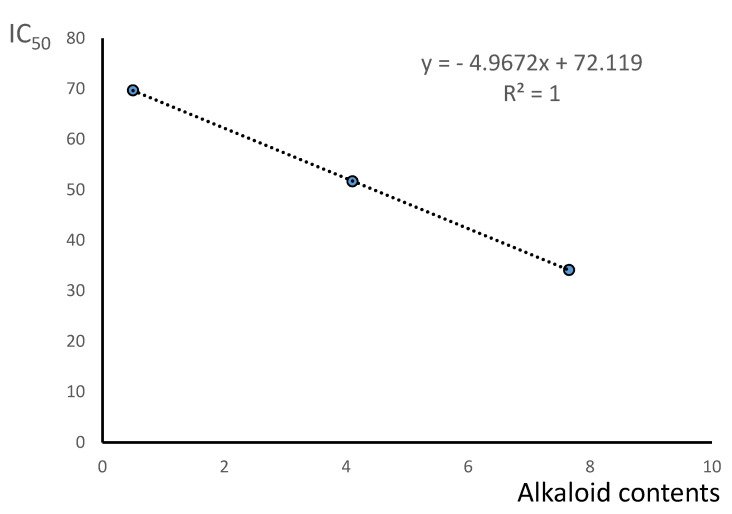
Correlation between contents of berberine and palmatine in *M. aquifolium* extracts obtained from various parts of plant collected during flowering with cytotoxic activity against A375 cell line.

The content of alkaloids, which showed strong cytotoxic properties (berberine and palmatine) in extracts obtained from various parts of *M. aquifolium*, was also correlated with the IC_50_ values obtained for these extracts (Figure 11 and Table 4). High values of the correlation coefficients were obtained for both the content of individual alkaloids and their total content. In most cases r values were higher than 0.98. Only content of berberine and content of sum of berberine and palmatine poorly correlated with cytotoxicity of *M. aquifolium* leaves extract against SK-MEL-3 cell line. On SK-MEL-3 cell line relatively high cytotoxic activity was observed for extract obtained from leaves which contain small amounts of determined alkaloids possessing high cytotoxic activity. A relatively lower correlation was also obtained between the palmatine content in extracts from various parts of the plant and its activity against A375 cells. The highest r values were obtained between berberine and sum of berberine and palmatine contents and IC_50_ values obtained on A375 cell line r = 0.9966 and 1.000, respectively. The higher r values obtained in most cases can indicated on very significant influence of isoquinoline alkaloids palmatine and especially berberine on cytotoxic properties of *M. aquifolium* extracts. The investigated extracts also contain other alkaloids which may also influence on their cytotoxic properties. For this reason, further research on both the composition and related cytotoxic properties are advisable.

## 3. Experimental

### 3.1. Chemicals and Plant Materials

Acetonitrile (MeCN), methanol (MeOH), 1-butyl-3-methylimidazolium tetrafluoroborate of chromatographic quality were obtained from E. Merck (Darmstadt, Germany), dimethyl sulfoxide (DMSO) was from Sigma-Aldrich (Saint Louis, MO, USA).

Alkaloid standards (magnoflorine and palmatine) were purchased from Chem Faces Biochemical Co. Ltd. (Wuhan, China). Berberine was purchased from Sigma-Aldrich (St. Louis, MO, USA).

Plant material was collected and identified in the Botanical Garden of Maria Curie-Skłodowska University in Lublin (Poland) in spring, summer, and autumn 2019. Voucher specimens of plant materials No. 12/2021 (see Supplementary Materials) was deposited in the Botanical Garden of Maria Curie-Skłodowska University in Lublin (Poland). Plant materials were identified by Grażyna Szymczak from Botanical Garden of Maria Curie-Skłodowska University in Lublin. Plant materials were collected between April and October 2020.

Plants were divided into roots, cortex, leaves, barked stalk, and fruit. Plants organs were cut into pieces and dried at ambient temperature for 1–2 weeks.

### 3.2. Apparatus and HPLC Conditions

#### 3.2.1. HPLC-DAD

Analysis was performed using an LC-20AD Shimadzu (Shimadzu Corporation, Canby, OR, USA) liquid chromatograph equipped with Synergi Hydro RP 80A (150 mm × 4.6 mm, 5 μm) and Synergi Polar RP 80A (150 mm × 4.6 mm, 5 μm) columns. The chromatograph was equipped with a Shimadzu 364 SPD-M20A detector (Shimadzu Corporation, Canby, OR, USA). Detection was carried out at a wavelength of 240 nm. All chromatographic measurements were controlled by a CTO-10ASVP thermostat (Shimadzu Corporation, Canby, OR, USA). The eluent flow rate was 1.0 mL/min. Extracts were injected into the columns using the Rheodyne 20 μL injector. The DAD detector was set in the 200–800 nm range. Data acquisition and processing were carried out with a LabSolutions software (Shimadzu Corporation, Kyoto, Japan). Analysis was performed on Synergi Polar RP 80A column (150 × 4.6 mm, 5 μm). The mobile phase was composed out of 0.04 ML^−1^ 1-butyl-3-methylimidazolium tetrafluoroborate in water (solvent A) and 1-butyl-3-methylimidazolium tetrafluoroborate in acetonitrile (solvent B) in gradient elution: 0–20 min, 25% B; 20–30 min, 25–32% B; 30–40 min, 32–40% 373 B, 40–60 min, 40% B. Flow rate was 1 mL/min.

Calibration curves were constructed by analyzing the alkaloid standards at eight concentrations, ranging from 0.001 to 0.2 mg/mL. The calibration curves were obtained by means of the least square method. The limit of detection (LOD) and limit of quantification (LOQ) obtained for alkaloids were calculated according to the formula: LOD = 3.3 (SD/S), and LOQ = 10 (SD/S), where SD is the standard deviation of response (peak area) and S is the slope of the calibration curve.

HPLC analyses of alkaloid standards and plant extracts were repeated three times.

#### 3.2.2. HPLC-MS/MS

Determination and identification of selected alkaloids was carried out using an HPLC Agilent 1290 Series system (Agilent Technologies, Germany) equipped with an ESI interface, a 6540 UHD accurate mass Q-TOF detector and Mass Hunter software for data collection and instrumental control. Chromatographic XDB-C18 column (4.6 mm × 50 mm, 1.8 μm, Agilent Technologies, Germany) was maintained at 20 ± 0.5 °C. The injected sample volume was 20 μL, while the mobile phase was composed ACN + 0.1% HCOOH (30:70) dosed at a flow rate of 0.6 mL/min. Quadrupole time-of-flight mass spectrometric analyses were performed using electrospray ion source operating in positive ion mode (ESI(+)), with the following set of operation parameters: capillary voltage (CV), 3.5 kV; octopole voltage (OV), 800 V; skimmer voltage (SV), 50 V; drying gas temperature (DGT), 295 °C; shielding gas temperature (SGT), 315 °C; mass spectra were recorded across the range mass range 40–370 *m*/*z*; fragmentor voltage (FV) 195 V. Nitrogen was used as drying (7 L/min) and nebulizng (40 psig) gas. The HPLC–MS data were acquired and quantified with the use of MassHunter Workstation software. The data were further processed using Microsoft Excel. The instrument was operated in extracted ion chromatogram (EIC) and product ion modes, respectively.

### 3.3. Extraction Procedure

The previously described procedure of alkaloids extraction from plant materials was applied after minor modifications [36,37]. 

Weighted Samples (5 g) of each plant were macerated with 100 mL ethanol for 72 h and continuously extracted in an ultrasonic bath for 5 h. Extracts were filtered, the solvent evaporated under vacuum, and the residues dissolved in 30 mL of 2% sulfuric acid and defatted with diethyl ether (3 × 40 mL). The aqueous layers were subsequently basified with 25% ammonia to a pH of 9.5–10 and the alkaloids extracted with chloroform (3 × 50 mL). After evaporation of the organic solvent, the dried extracts were dissolved in 5 mL MeOH prior to HPLC analysis. 

### 3.4. Investigation of Cytotoxic Activity

Cytotoxicity of the plant extracts, magnoflorine, palmatine and berberine were examined against a panel of three melanoma cell lines (A375, G-361, SK-MEL-3) characterized by different degrees of genetic complexity. The investigated cell lines were obtained from American Type Culture Collection (ATCC; Manassas, VA, USA). A375 cells were cultured in Dulbecco’s Modified Eagle’s Medium (DMEM) (Sigma Aldrich, St. Louis, MO, USA) supplemented with 10% heat inactivated fetal bovine serum (FBS), penicillin (100 U/mL), and streptomycin (100 µg/mL). Human melanoma SK-MEL-3 and G-361 cells were maintained in McCoy’s 5A Medium (Sigma Aldrich, St. Louis, MO, USA) supplemented with 15% (for SK-MEL-3) or 10% (for G-361) FBS, penicillin (100 U/mL), and streptomycin (100 μg/mL). The cells were maintained at 37 °C in a 5% CO_2_ atmosphere. The dried plant extracts and standards (magnoflorine, palmatine, berberine) were dissolved in DMSO in order to obtain stock solutions at concentrations of 50 mg/mL. At the day of experiment, the suspension of cells (1 × 105 cells/mL) in respective medium was applied to a 96-well plate at 100 μL per well. After 24 h of incubation, the medium was removed from the wells and replaced by increasing concentrations of plant extracts or standards in medium containing 2% FBS. The control cells were only cultured with a medium containing 2% FBS. Cytotoxicity of DMSO was also checked at concentrations present in respective dilutions of stock solutions. After 24 h incubation, 15 μL MTT working solution (5 mg/mL in PBS) was added to each well. The plate was incubated for 3 h. Subsequently, 100 μL of 10% SDS solution was added to each well. Cells were incubated overnight at 37 °C to dissolve the precipitated formazan crystals. The concentration of the dissolved formazan was evaluated by measuring the absorbance at λ = 570 nm using a microplate reader (Epoch, BioTek Instruments, Inc., Winooski, VT, USA). Two independent experiments were performed in triplicate. The results of the MTT assay were expressed as mean ± SD. DMSO in the concentrations present in the dilutions of stock solutions did not influence the viability of the tested cells. IC_50_ values of the investigated extracts and alkaloid standards were calculated using IC_50_ calculator (www.IC50.tk (accessed on 22 January 2021)).

## 4. Conclusions

All tested plant extracts contained isoquinoline alkaloids with cytotoxic activity. Large differences were obtained in the content of alkaloids in extracts obtained from various parts of the plant as well as in extracts obtained from the plant material collected in various vegetation seasons. In roots of *Mahonia aquifolium* collected during flowering a large amount of berberine (above 3.3 mg/g of plant material), palmatine (0.79 mg/g of plant material) were determined.

The highest activity against all tested cancer cell lines was found for berberine. Palmatine also showed high cytotoxicity, while magnoflorine showed only slight cytotoxic activity.

Our data demonstrated that the extracts obtained from different parts of *Mahonia aquifolia* have very high cytotoxic activity against A375, G361, and SKMEL3 cancer cell lines. To the best of our knowledge, the cytotoxic activity of the extracts has not been yet investigated against tested by us cell lines.

Very good correlations were obtained between contents of determined alkaloids possessing high cytotoxic activity (berberine and palmatine) with cytotoxicity of investigated plant extracts obtained from various parts of plant.

Higher cytotoxicity was found for extracts containing highly concentration of cytotoxic alkaloids berberine and palmatine The highest cytotoxic activity against all tested cancer cell lines was observed after applying the *Mahonia aquifolium* roots and cortex extracts.

The differences between the cytotoxic activities of the different parts of investigated plants strongly depend on alkaloids content and a synergic effect of the different alkaloids may be influence on extract activities.

Based on these results, investigated plant extracts especially obtained from *Mahonia aquifolium* root and cortex collected during flowering can be recommended for further in vivo experiments. Investigated extracts and alkaloids contained therein may be developed as a new candidate anticancer agent for human malignant melanoma.

## Figures and Tables

**Table 1:** Retention times and equation of calibration curve, correlation coefficients (r), limit of detection (LOD) and limit of quantification (LOQ) values for studied isoquinoline alkaloids.

Alkaloid	t_R_	Equation of Calibration Curve	r	LOD [mg/mL]	LOQ [mg/mL]
Berberine	32.95	*y* = 70,984,852*x* − 3,300,769	0.9990	0.0128	0.0422
Magnoflorine	4.42	*y* = 25,635,805*x* − 251,782	0.9998	0.0039	0.0120
Palmatine	28.55	*y* = 52,900,150*x* + 732,112	0.9982	0.0009	0.0028

**Table 2:** Contents of isoquinoline alkaloids in plant extracts.

Alkaloid	Contents of Alkaloids in Plant Extracts Obtained from Different Parts of *Mahonia aquifolium* Collected in Various Vegetation Seasons (mg/g of Dry Plant Material)		
	Before Flowering	During Flowering	After Flowering
Cortex			
Berberine	0.133 *	3.313	0.066
Magnoflorine	0.086 *	-	0.099
Palmatine	0.036 *	0.790	0.789
Leaves			
Berberine	-	0.0217	<LOQ
Magnoflorine	0.322 *	0.2342	0.317
Palmatine	-	<LOQ	-
Barked stalk			
Berberine	●	0.436	0.043
Magnoflorine	●	-	0.188
Palmatine	●	0.065	0.339
Roots			
Berberine	●	7.036	0.0074
Magnoflorine	●	-	-
Palmatine	●	0.620	0.299
Fruit			
Berberine	●	●	<LOQ
Magnoflorine	●	●	-
Palmatine	●	●	-

*: Data published in ref [29]; -: peak was not identified; <LOQ: below limit of quantification; ●: The extract was not investigated.

**Table 3:** Cytotoxic activity, expressed as IC_50_ values, of the investigated extracts and alkaloid standards against three melanoma cell lines (A375, G361, SK-MEL-3).

	IC_50_ (µg/mL)		
	A375	G361	SK-MEL-3
*Mahonia aquifolium* root	34.13 ± 0.41	5.56 ± 1.07	10.53 ± 0.12
*Mahonia aquifolium* cortex	51.66 ± 1.61	4.78 ± 0.78	16.80 ± 0.26
*Mahonia aquifolium* leaves	>200	15.55 ± 2.04	11.50 ± 0.75
*Mahonia aquifolium* barked stalk	69.67 ± 1.66	14.49 ± 1.76	41.66 ± 0.89
Berberine	52.73 ± 5.63	21.25 ± 3.25	40.94 ± 5.42
Magnoflorine	>200	>200	>200
Palmatine	>200	119.98 ± 10.44	88.04 ± 7.16

**Table 4:** Equation of correlation curve and correlation coefficients (r), values between isoquinoline alkaloid contents and cytotoxic activity expressed as IC_50_ of investigated plant extracts.

Cell Line	Berberine		Palmatine		Berberine and Palmatine	
	Equation	r	Equation	r	Equation	r
A375	y = −5.35x + 71.06	0.9966	y = −34.55x + 68.81	0.7372	y = −4.97x + 72.12	1.0000
G361	y = −1.52x + 14.286	0.8490	y = −14.06x + 15.19	0.9882	y = −1.42x + 14.45	0.8889
SK-MEL-3	y = −2.20x + 26.07	0.4877	y = −39.69x + 42.51	0.9140	y = −2.01x + 26.30	0.4899

## Data Availability

Data is contained within the article or supplementary material.

## Supplementary Materials

The following are available online. Figure S1: Voucher specimens of plant materials No. 12/2021.
