# Supplementary figures and images for: Determination of Cytotoxic Activity of Selected Isoquinoline Alkaloids and Plant Extracts Obtained from Various Parts of Mahonia aquifolium Collected in Various Vegetation Seasons
Source: Molecules. 2021 Feb 4;26(4):816. doi: 10.3390/molecules26040816 (PMC7915140; doi:10.3390/molecules26040816)

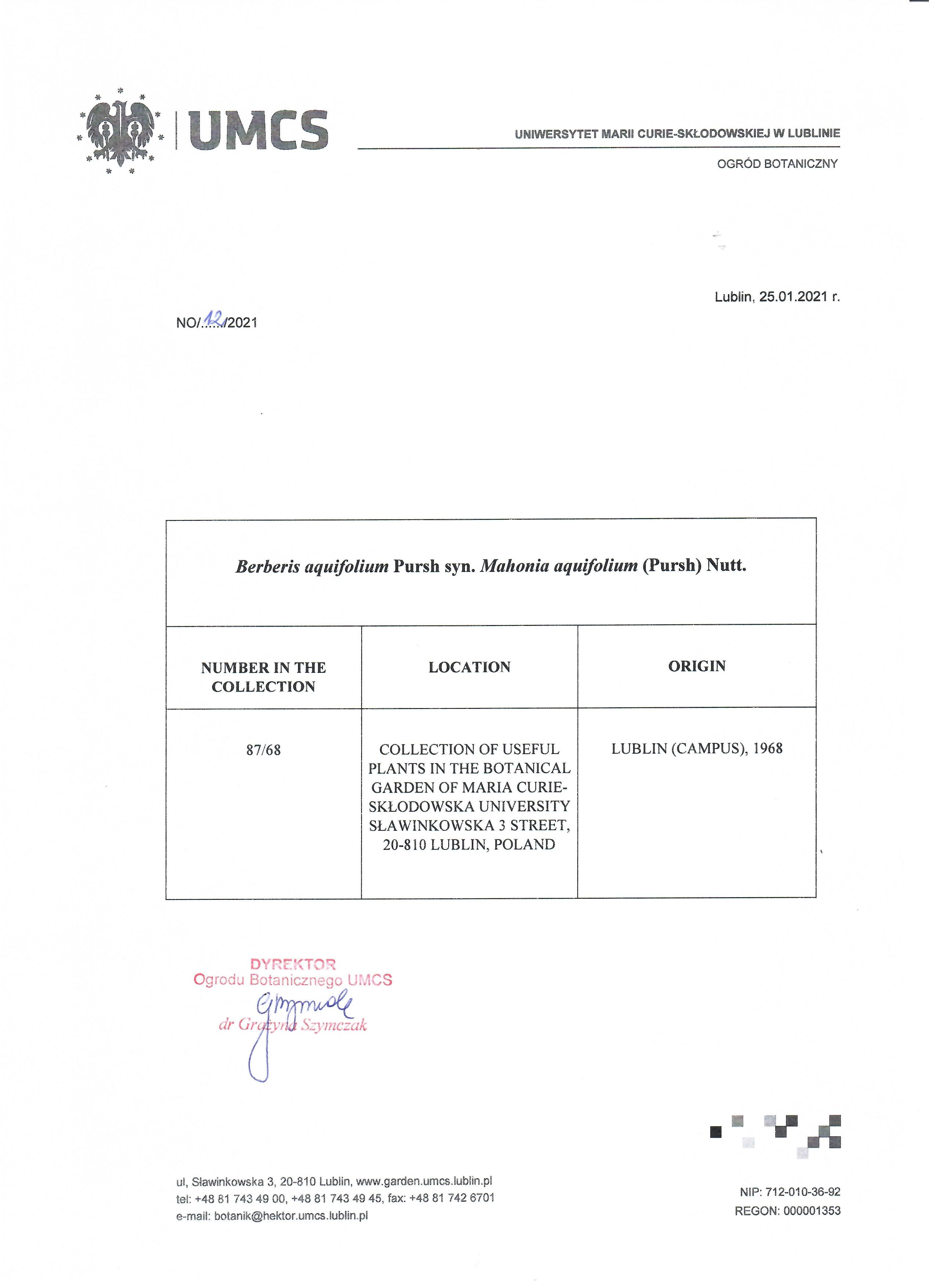

Supplement: Supplementary file 1 [file molecules-26-00816-s001.zip › molecules-1089328-supplementary.png]
